# Integration of a Smartphone HF-Dedicated App in the Remote Monitoring of Heart Failure Patients with Cardiac Implantable Electronic Devices: Patient Access, Acceptance, and Adherence to Use

**DOI:** 10.3390/jcm12175528

**Published:** 2023-08-25

**Authors:** Matteo Ziacchi, Giulio Molon, Vittorio Giudici, Giovanni Luca Botto, Miguel Viscusi, Francesco Brasca, Amato Santoro, Antonio Curcio, Michele Manzo, Erminio Mauro, Mauro Biffi, Alessandro Costa, Andrea Dell’Aquila, Maria Carla Casale, Giuseppe Boriani

**Affiliations:** 1 Istituto di Cardiologia, IRCCS Azienda Ospedaliero Universitaria di Bologna, Via Massarenti 9, 40138 Bologna, Italy; matteo.ziacchi@gmail.com; 2 IRCCS Sacro Cuore Don Calabria, 37024 Negrar, Italy; giulio.molon@sacrocuore.it (G.M.); alessandro.costa@sacrocuore.it (A.C.); 3 Cardiologia Riabilitativa, ASST Bergamo EST, 24068 Seriate, Italy; vittorio.giudici@asst-bergamoest.it; 4 ASST Rhodense, 20020 Rho, Italy; gluca.botto@gmail.com (G.L.B.); mariacarlacasale@gmail.com (M.C.C.); 5 AORN S. Anna e S. Sebastiano, 81100 Caserta, Italy; mg.viscusi@gmail.com; 6 Department of Cardiovascular Neural and Metabolic Sciences, San Luca Hospital, IRCCS Istituto Auxologico Italiano, 20095 Milano, Italy; fmabrasca@gmail.com; 7 AOU Senese, 53100 Siena, Italy; amato.santoro@gmail.com; 8 AOU Mater Domini, 88100 Catanzaro, Italy; curcio@unicz.it; 9 AOU S. Giovanni di Dio e Ruggi d’ Aragona, 84131 Salerno, Italy; michele.manzo@sangiovannieruggi.it; 10 Policlinico di Modena, AOU Modena, 41125 Modena, Italy; erminio.mauro@alice.it; 11 Elettrofisiologia e Aritmologia, ASST Bergamo EST, 24068 Seriate, Italy; andrea.dellaquila@hotmail.com; 12 Cardiology Division, Department of Biomedical, Metabolic and Neural Sciences, University of Modena and Reggio Emilia, Policlinico di Modena, 41124 Modena, Italy; giuseppe.boriani@unimore.it

**Keywords:** heart failure management, smartphone app, integrated diagnostics, patient-centered care, telemedicine

## Abstract

(200 w) Introduction. Remote monitoring (RM) of cardiac implantable electronic device (CIED) diagnostics helps to identify patients potentially at risk of worsening heart failure (HF). Additionally, knowledge of patient HF-related symptoms is crucial for decision making. Patient smartphone applications may represent an ideal option to remotely collect this information. Purpose. To assess real-world HF patient access, acceptance, and adherence to use of an HF-dedicated smartphone application (HF app). Methods. In this study, 10 Italian hospitals administered a survey on smartphone/app use to HF patients with CIED. The subgroup who accepted it downloaded the HF app. Mean 1-year adherence of the HF app use was evaluated. Results. A total of 495 patients (67 ± 13 years, 79% males, 26% NYHA III–IV) completed the survey, of which 84% had access to smartphones and 85% were willing to use the HF app. In total, 311/495 (63%) downloaded the HF app. Patients who downloaded the HF app were younger and had higher school qualification. Patients who were ≥60 years old had higher mean 1-year adherence (54.1%) than their younger counterparts (42.7%; *p* < 0.001). Hospitals with RM-dedicated staff had higher mean 1-year patient adherence (64.0% vs. 33.5%; *p* < 0.001). Adherence to HF app decreased from 63.3% (weeks_1–13) to 42.2% (weeks_40–52, *p* < 0.001). Conclusions. High access and acceptance of smartphones/apps by HF patients with CIED allow HF app use for RM of patient signs/symptoms. Younger patients with higher school qualifications are more likely to accept HF app; however, older patients have higher long-term adherence.

## 1. Introduction

Heart failure (HF) is a serious cardiovascular condition associated with significant morbidity, mortality, and healthcare costs. Currently, the incidence of HF in Europe is about 3/1000 person years (all age groups) or about 5/1000 person years in adults [1].

The most relevant determinants of HF-related costs are acute heart failure (AHF) hospitalizations [2]. Despite therapy optimization, more than 25% of patients are readmitted to hospital due to recurrence of AHF within 30 days of discharge, mainly due to congestion [3]. Due to population growth, ageing, and the increasing prevalence of comorbidities, the absolute number of hospital admissions for HF is expected to increase by 50% in the next 25 years [1]. Clinical signs of initial congestion, such as left ventricular filling pressure elevation, subclinical fluid overload, weight gain, and blood pressure changes [4], appear days or weeks before AHF hospitalization Therefore, in recent years, many strategies for early detection of subclinical and potentially actionable impending AHF events have been investigated in an attempt to mitigate individual patient risk of hospitalization. In this context, remote monitoring may be a valuable approach for HF patients implanted with a cardiac electronic device (CIED) by virtue of CIED’s ability to detect real-time changes in physiological parameters that may precede AHF events (i.e., heart rhythm, heart rate, intrathoracic fluid status, and activity) [5,6]. Additionally, remote monitoring can provide clinicians with a HF risk status based on a number of CIED-collected diagnostic parameters [7]. By means of validated multiparametric analysis, CIEDs from different manufacturers can estimate the risk of developing AHF in the subsequent month, thus allowing physicians to act at a preclinical or subclinical stage. In particular, the HF risk score (HFRS) provided by the Medtronic TriageHF^TM^ algorithm has been validated for AHF prediction on a large cohort of patients implanted with ICD or CRT-D devices [7,8,9] and has also been shown to predict all-cause and non-HF-related cardiovascular hospitalizations [9,10] as well as all-cause mortality [11].

While AHF prediction can potentially mitigate individual patient risk of hospitalization, CIED data and HFRS only indicate patients potentially at risk of an acute cardiovascular event, without providing useful indication related to the type and timing of clinical action that should be taken. Patient smartphone applications (apps) may represent an ideal option to collect information about patient HF-related signs, symptoms, and behaviors [12] and integrate automatic HFRS data to support clinicians in the decision-making process. Although some patient surveys on the adoption of smartphones and use of mobile health apps have been previously published [13,14,15,16], they are limited to single-center experiences conducted in the United States (US), including small samples of HF patients [13,14,15] or patients with CV disease (not specifically HF) or CV risk factors [16], and none refer to HF population with CIED from out-of-US (OUS) geographies.

The purpose of the Angels of HF project is to assess the real-world benefit of including a patient app to collect information on HF symptoms, signs, and therapy adherence and the new HF risk score [7] based on CIED-recorded information for the remote management of HF patients with CIED. The first phase of the project, the results of which are reported in the present manuscript, focused on assessing the following endpoints in a large, real-world Italian HF population with CIED: (1) patient ownership or access to smartphone/tablet and app technology and reasons for not owning a smartphone/tablet; (2) patients’ or caregivers’ acceptance to receive an HF-dedicated app to send a weekly diary on HF signs, symptoms, and therapy adherence to their reference site as well as the characteristics of those who downloaded and activated the app (app group) in comparison to those who had not received the app (no app group); and (3) mean patient adherence to using the app during the first year of usage (app group only).

## 2. Methods

Angels of HF is an ongoing project that includes patients with a diagnosis of heart failure wearing a Medtronic implantable cardioverter defibrillator (ICD) and/or cardiac resynchronization therapy (CRT) device equipped with automatic alerts for lung fluid accumulation (OptiVol^®^) and a CareLink monitor for scheduled remote device checks. Angels of HF is part of One Hospital Clinical Service (OHCS), a larger medical care project provided by Medtronic to hospitals under a signed agreement, that includes technical and statistical support for collection, management, analysis, and reporting of real-world data from patients treated with Medtronic therapies. The aim is to describe and improve the quality of diagnostic and therapeutic strategies using technologies/therapies in clinical practice. As part of OHCS, participating centers are provided with (1) access to a website for data collection and review; (2) the possibility to provide HF patients with an HF-dedicated patient app to periodically collect data on HF symptoms, signs, and therapy adherence; (3) data management; (4) periodic reports on merged clinical, CIED, and app data; and (5) upon physicians’ request, ad-hoc statistical analyses. The project and related data-treatment activities were approved by the Ethical Committee or the relevant Institutional Review Boards of each institution and conform to the ethical guidelines of the 1975 Declaration of Helsinki. Each patient signed an informed consent for participation.

From January 2021, all consecutive HF patients with Medtronic devices who accessed the cardiology department of the participating sites for routine follow-up visits were approached by the project physician or project coordinator, who provided information about the Angels of HF project’s aim and procedures, including a short description and the purpose of the HF-dedicated app and asked patients to sign the informed consent if they decided to participate.

### 2.1. Survey Design and Collection

In the first phase of the project, from January 2021 to July 2022, patients who agreed to participate were asked to respond to a 10-question survey to evaluate the following: access to a smartphone or a tablet, ability in using such technology and apps, previous experience with health apps, and willingness to use an HF-dedicated app to send periodic information to the reference site. The survey was designed by two physicians leading the project and reviewed by the physicians or coordinators from participating sites. Compared with other published surveys [15,17,18], the number of questions was reduced to a one-page survey with the intent of increasing the completion of all sections. Only questions most strictly related to the project aims were included in the survey. In addition, the collection of caregiver’s answers to the survey was introduced for inclusion of patients who do not own a smartphone but have a caregiver owning one. The survey was designed to be completed by all patients or their caregivers while waiting in the hospital waiting room prior to a follow-up visit or, alternatively, with the help of the project physician/coordinator before starting the visit. The final version of the survey was tested with the first 3 HF patients of the coordinator center, and after confirmation of the full understanding and quickness of completion (less than 5 min), it was released for use in the project. All answers were captured on paper and then transcribed by the project site personnel into the OHCS web-based platform. Appendix A reports all questions from the survey.

Patient demographics, school qualification, medical history, baseline assessment (NYHA class, LVEF, and QRS morphology and duration), baseline medications, and CIED information were also collected in the OHCS platform. During follow-up, all AHF events, and hospitalizations, all applied therapies and change in medications were collected in the same platform.

### 2.2. Patient HF-Dedicated App Description

**Figure 1:**
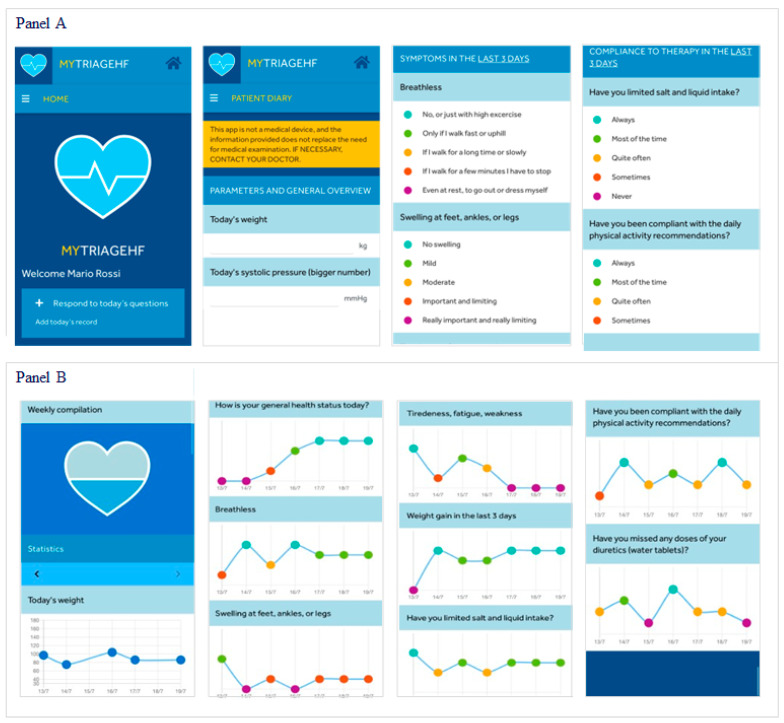
MyTriageHF app layout: example of some app screenshots of the English version. Italian patients were provided with the Italian version of the app. Panel (**A**) showes the desktop of the App with some information collected in the patient diary and questions about HF symptoms of the last days; Panel (**B**) showes the trends of the answers reported by the patient on the health status, symptoms and therapy compliance.

MyTriageHF app (Figure 1) is a native app for smartphones or tablets designed by Medtronic in close collaboration with all physicians involved in the Angels of HF project to remotely collect patient HF signs, symptoms, and adherence to medical prescriptions. The MyTriageHF app data can be integrated with information received through the remote monitoring system from CIED diagnostic parameters whose variation may precede an episode of AHF (thoracic impedance, fluid index, heart rate, heart rate variability, patient activity, ventricular and atrial pacing percentage, atrial and ventricular arrhythmia burden, and multiparametric HFRS) to improve their specificity and help physicians in remotely deciding whether, when, and which therapeutic intervention is needed to avoid patient HF hospitalization.

All patients provided with the app are able to fill in a daily patient diary composed of 11 questions. These 11 questions include collection of weight and systolic and diastolic blood pressure as well as 8 questions formatted as a 5-point Likert scale related to general health status; HF-related signs and symptoms experienced during the last 3 days (breathless; swelling at feet, ankles, or legs; fatigue; and weight gain); and patient’s adherence to medical prescriptions in terms of limitation of salt and liquid intake, physical activity, and diuretic intake (Figure 1, panel A). If some of the 8 multiple-choice questions are not answered, a message for completion appears, and the diary cannot be saved and sent until completed. Within the app, every patient has the possibility to review transmitted diaries and trending responses throughout time (Figure 1, panel B). Additionally, each patient will have access to educational materials (videos and brochures related to educational heart failure information and life with a cardiac implantable device) and the possibility to annotate the name and phone number of hospital contacts and the date of next follow-up. The HF-dedicated app does not allow direct communication with hospital personnel or receipt of automatic reminders for completing the diaries, symptom management tips, or any reminders on medication/exercise.

In the first phase of the project, the app was available free of charge only on Google Play or Apple Store.

In the OHCS web-based platform, the physicians or hospital-delegated trained personnel can access all received patient diaries as well as a patient report, which is updated on a weekly basis for each HF patient. The weekly patient report includes patient adherence to the app usage during the last 6 months as well as the previous 2-month trends of HFRS, patient diary responses, and all the device diagnostics whose variation may precede an AHF event. In case of an AHF event, the event date and information related to an increase in diuretic therapy are also marked in the trends. In this study, the timing for review of patient diaries or the patient report and the decision to provide feedback or advice on clinical actions to patients was left to physician discretion. The patients were informed of the app not being a medical tool or an emergency alert system upon signing the informed consent and during the training on the use of the app. In addition, a highlighted message was reported in the app at the beginning of each new diary, stating “this app is not a medical device and the information provided does not replace the need for medical examination. IF NECESSARY, CONTACT YOUR DOCTOR”.

### 2.3. Patient HF-Dedicated App Download and Training

The patients willing to use the app were provided with credentials to log in by the project physician or coordinator. Subsequently, patients were assisted in downloading the app from the app store on their smartphone or tablet, trained on the use of the app, and supported in sending their first diary through the app. The patients were not provided with incentives to participate in the project or for use of the app. During the training, which took around 20 min/patient, the project physician or coordinator explained to the patient and/or the caregiver that the app was designed with the aim of collecting additional data on patient condition during follow-up to be integrated with other available CIED data in order to improve HF patient management. They also clearly specified that the information provided with the app was not intended to replace the need for medical examination and that in case of impaired clinical condition, they should contact their doctor. Detailed written instructions, including app screenshots, were provided to patients or their caregiver for further information. Patients were provided technical support by the project coordinator or site-delegated personnel throughout the study for lost login information, changes in phone devices, and/or other well-known issues that can affect access and use.

All patients who agreed to use the app were asked to fill in at least one diary per week.

### 2.4. Statistical Analysis

Descriptive statistics were used to summarize all results. These included mean and standard deviation or median with interquartile range (IQR) for continuous variables as well as counts and percentages for categorical variables. Continuous variables were compared between groups using Wilcoxon’s test or Kruskal–Wallis test as appropriate. Categorical variables were compared between groups using the chi-square test or Fisher’s exact test as appropriate. Categorical variables within groups were compared using McNemar’s test.

Mean 1-year adherence to using the app was calculated as the percentage of weeks in a year with 1 or more diaries sent through the app by all patients having the app for at least one year. Mean adherence was also calculated for each quarter period (weeks 1–13, weeks 14–26, weeks 27–39, and weeks 40–52). Patients who accepted and activated the app on their own or their caregiver’s smartphone/tablet were included in the “app group”, while the rest of the patients were included in the “no app group”. Based on the availability of dedicated site personnel to follow up HF patients with remote monitoring, participating sites were a priori defined as “high-organized sites” if they had dedicated staff for remote monitoring of HF patients or as “low-organized sites” if they did not.

Based on the number of patients who followed through the HF-dedicated app at the time of statistical analysis, participating sites were considered “low-usage sites” if they had ≤20 patients (in the low 20% of the distribution) using the app, “mid-usage sites” if they had 21–30 patients (within 21–60% of the distribution) using the app, and “high-usage sites” if they had ≥30 patients (over 61% of the distribution) using the app.

All statistical tests were based on a two-sided significance level of 0.05.

SAS software, version 9.4 (SAS Institute Inc., Cary, NC, USA), was used to perform statistical analyses.

## 3. Results

**Figure 2:**
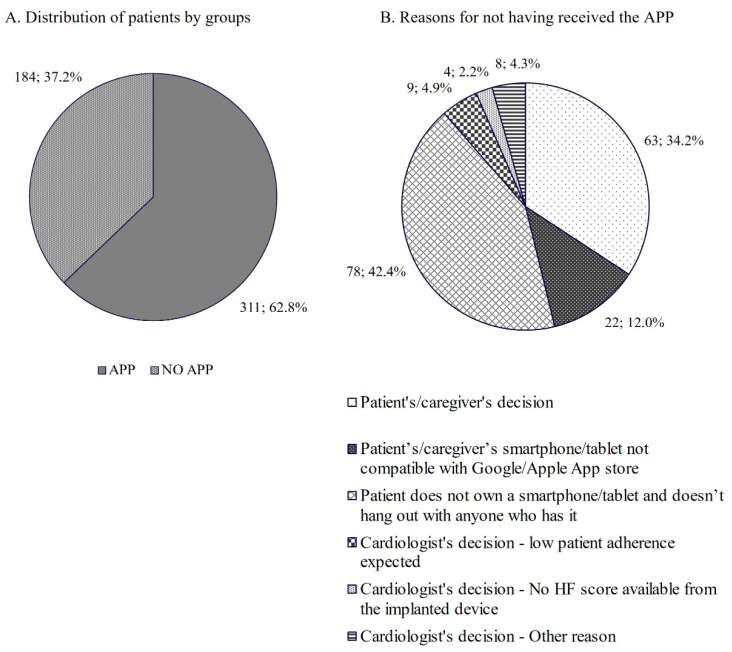
Distribution of patients by app and NO app groups (panel (**A**)) and reasons for not having received the app (panel (**B**)).

From January 2021 to July 2022, 495 consecutive HF patients with CIED (age 67 ± 13 years, 79% males, 26% NYHA III-IV, LVEF 35 ± 11%, 60% with three-chamber CIED) accessed the cardiology department of 10 Italian hospitals for standard in-hospital follow-up. All of them agreed to participate in the project and completed the 10-question survey on the use of smartphone/tablet and app technologies. Out of the 495 patients who completed the survey, 311 patients (62.8%) agreed to receive and downloaded the HF-dedicated app on their or their caregiver’s smartphone (app group), while 184 were not provided with the app (NO app group) (Figure 2, panel A). Out of the 184 NO app patients, 63 (34.2%) refused to receive it based on their personal or their caregiver’s decision; other reasons for not downloading the app included (1) not owning a smartphone/tablet or no relationship with anyone owning a smartphone/tablet for 78 (42.4%) patients, (2) incompatibility of the patient’s/caregiver’s smartphone/tablet with Google Play/Apple Store to download the app for 22 (12.0%) patients, and cardiologist’s decision for the remaining 21 (11.4%) patients (Figure 2, panel B).

Patient demographics, medical history, device type, and baseline medications are described in Table 1. The app group was younger and had a higher percentage of patients with RBBB than the NO app group (Table 1). No other difference in baseline characteristics was observed.

### 3.1. Survey Results

Out of the 495 patients, 417 (84.2%) had access to a smartphone or a tablet directly (298 patients, 60.2%) or through a cohabiting (70 patients, 14.1%) or non-cohabiting caregiver (49 patients, 9.9%). The main reasons for not owning a smartphone or a tablet among 197 patients were as follows: not being used to this technology (129 patients; 65.5%), smartphone/tablet considered unnecessary (64 patients; 32.5%), and not knowing what a smartphone is (19 patients; 9.6%). On the other hand, smartphone or internet connection costs were not considered a barrier (2.0% and 0.5% of respondents, respectively).

Looking at the subgroup of 448 patients/caregivers who provided information about their school qualification, 248 (55.3%) reported a low school qualification (primary or lower secondary school), 190 (42.4%) had a high school qualification (high school, bachelor’s degree, or master’s degree), while 10 (2.2%) had other qualifications. A higher percentage of patients with high school qualification (51.6%) belonged to the app group compared to the NO app group (23.3%, *p* < 0.001).

Among the 417 patients with access to a smartphone/tablet, 352 patients/caregivers (84.4%) had been using a smartphone for more than 2 years, with no difference between the app and NO app groups (*p* = 0.554). A total of 61.6% of patients/caregivers reported their ability to perform all high-level activities with their smartphone (app use, app installation, send an email, and browse the web). In particular, 286 (68.6%) patients/caregivers were able to use apps and 257 (61.6%) to install apps. The app group was more confident in the use of smartphone technology in general (71% able in all high-level activities) than the NO app group (34%; *p* < 0.001). Moreover, in comparison to the NO app group, the app group was also more able to install apps (71.4% in app group vs. 33.0% in NO app group; *p* < 0.001) and use apps (79.4% in app group vs. 36.8% in NO app group; *p* < 0.001). A total of 89 patients/caregivers (21.4% of the respondents) had already used health apps, and it was more often the case in the app group (77 subjects; 24.8%) than in the NO app group (12 subjects; 11.4%; *p* < 0.001).

Of the 417 patients, 351 with access to a smartphone/tablet (84.8%) showed willingness to send information on HF status, symptoms, and adherence to medical prescriptions using a free HF-dedicated app on a weekly basis, although 27 (6.5%) were afraid to forget this task and 24 (5.8%) were not confident in finding someone able to help him/her in this activity. Out of the 63 subjects (15.1%, including all in the NO app group) who were not willing to use the HF-dedicated app, only 1 subject (1.5%) reported concerns in terms of potential data privacy violation, while a few of them (5 subjects, 7.9%) stated that they did not have time to use the app and the remaining 55 subjects (87.3%) did not provide any explanation.

### 3.2. Adherence in the Use of the HF-Dedicated App

**Figure 3:**
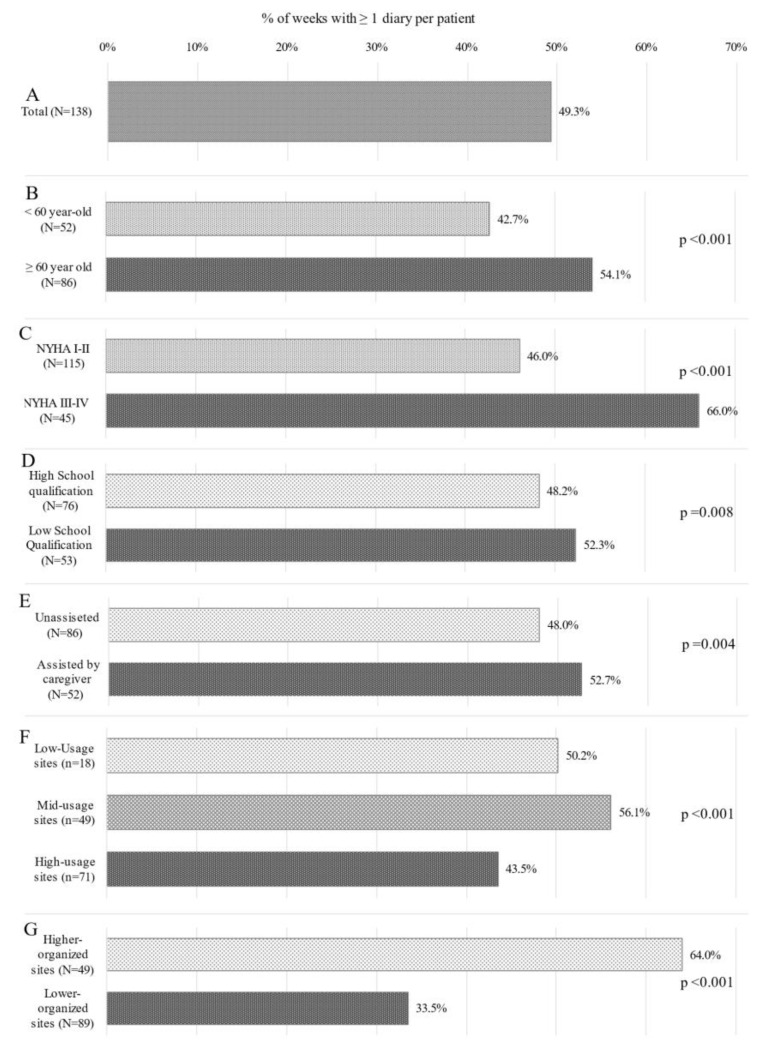
Mean 1-year patient adherence to the use of the HF-dedicated app, calculated as the percentage of weeks in a year with at list one completed diary sent through the app by all patients having the app for at least one year. (**A**) Adherence of total population, (**B**) Adherence based on age, (**C**) Adherence based on NYHA Class, (**D**) Adherence based on School Qualification, (**E**) Adherence based on need of assistance for app use, (**F**) Adherence based on the number of patients managed with the HF-dedicated app by the site, (**G**) Adherence based on level of site organization.

Among the 311 patients who downloaded the HF-dedicated app on their own or their caregiver’s smartphone (app group), 175 (56.3%) did not need any assistance in the app use on their own smartphone, while 136 (43.7%) needed some assistance to use it on their own (52; 16.7%) or their caregiver’s (84; 27.0%) smartphone. A total of 227 (73.0%) patients sent ≥2 diaries, 29 (9.3%) sent only one diary, while 55 (17.7%) of them did not send any diary. In July 2022, when the OHCS database was frozen for the present analysis, 138 (44.4%) subjects had the app for at least 1 year. Mean adherence of these patients in terms of app usage during the first year was 49.3% (Figure 3, panel A). Patients older than 60 years had a higher mean 1-year adherence (54.1%) than younger patients (42.7%; *p* < 0.001; Figure 3, panel B). Patients in NYHA class III–IV at baseline had higher mean 1-year adherence (66.0%) than patients with NYHA I–II (46.0; *p* < 0.001 Figure 3, panel C). Patients with lower school qualification had slightly higher mean 1-year adherence (52.3%) in comparison to those with higher school qualification (48.2%, *p* = 0.008 Figure 3, panel D). Patient assisted by a caregiver in the app use had slightly higher mean 1-year adherence than unassisted patients (52.7% vs. 48.0%; *p* = 0.004; Figure 3, panel E). Sites where the number of patients using MyTriageHF app was between 20 and 30 (mid-usage sites) had higher mean 1-year adherence (56.1%) than low-usage (50.2%) and high-usage (43.5%); *p* < 0.001 Figure 3, panel F) sites. High-organized sites achieved a higher mean 1-year patient adherence (64.0%) than low-organized sites (33.5%, *p* < 0.001 Figure 3, panel G).

**Figure 4:**
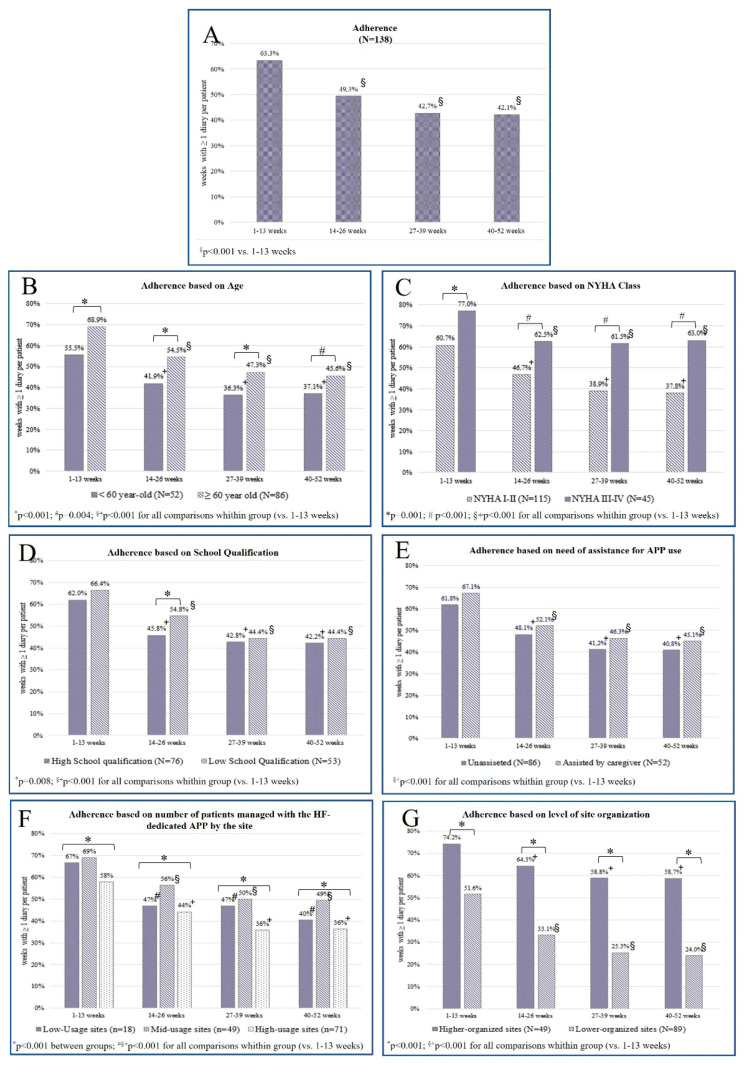
Mean patient adherence per quarter in the first year of the HF-dedicated app usage.

Mean adherence decreased with time from 63.3% (weeks 1–13) to 42.1% (weeks 40–52; *p* < 0.001; Figure 4), achieving a plateau after 26 weeks. Adherence decreasing with time was also noteworthy in the subgroups (Figure 4, panels B–G).

Table 2 summarizes the main findings of our project in comparison to other work in the field of smartphone/tablet app usage for HF management.

## 4. Discussion

Reduction in hospitalization, symptomatic improvement, and maximization of functional capacity represent the major challenges in heart failure management for the coming years. In HF patients with CIEDs, remote monitoring of implanted devices can help improve patient management through early diagnosis of heart failure events or risk stratification of cardiovascular and noncardiovascular adverse events [6,9,10,11]. To improve the specificity of CIED remote monitoring and its ability to guide clinical decision making, an app was designed for a patient smartphone or tablet to receive information about clinical state of HF patients during follow-up. Although multiple trials have shown the feasibility and benefits of different types of mobile-app-based interventions in recent years [19], OUS real-world data on patient access, acceptance, and adherence to actively using smartphone apps for HF management are currently limited [19,20,21], in particular for the subgroup of HF patient wearing a CIED (Table 2).

### 4.1. Smartphone/Tablet Technology Penetration

Our survey showed that in a large sample of Italian HF patients with CIED, more than 60% own a smartphone/tablet and an additional 24% have a caregiver able to provide it and assist in the app use. These results are aligned with the 2021 US data reporting that even if 97% of Americans own a mobile phone, only 85% of them own a smartphone, with lower percentages in the population older than 65 years (92% and 61%, respectively) [22]. High penetration of smartphone/tablet technology has also been previously reported by a big survey conducted in the US on patients with cardiovascular disease/risk factor [16] and by other small surveys on HF patients from single-center experiences [13,14,15] (Table 2). In 2021, due to the profound impact of the COVID-19 pandemic, mobile phone penetration in Europe increased up to 86% and smartphone adoption achieved 80% [23]. All smartphone owners are able to perform phone calls, and around 77% of them are mobile internet users (they surf the net or use internet-based messaging, social media, or other apps) [23]. Due to such high penetration, smartphone technology has already been explored as a tool to facilitate patient management both in heart failure [19] and other chronic diseases [24,25]. Our survey confirmed that smartphone app technology could be applicable to collect patient health status and therapy adherence in the HF management of patients with CIED as about 70% of patients or their caregivers are able to use smartphone apps, in line with European data.

### 4.2. Health App User Profile

Internet-based apps are more commonly used by young people [13,17,18]. Health apps are less used compared to the other apps (in the US, about 35% of adults with a smartphone/tablet [26] and 48% of those with a cardiovascular disease or risk factors [16] use a health app), but the adoption is rapidly increasing, despite a lack of clear legislation regulating the use [27]. In our HF cohort, younger patients with a higher school qualification most often agreed to use an app to improve HF disease management. This result is in line with another recent survey published by Boriani et al. investigating the option of telemedicine and digital therapy to implement medical practice [17] and also with previous US surveys [13,15] (Table 2). On the other hand, patients who are not willing to use the app are older with a lower school qualification. It is interesting to note that, unlike other reports on new technology acceptance by older adults [14,28,29], our survey showed that resistance to this type of technology is not driven by potential costs or data privacy fears but is mainly due to the lack of confidence in the use of the app.

### 4.3. The Role of Caregivers

A proactive patient role is fundamental in heart failure management [19,30]. In a study published more than 10 years ago in a different but comparable setting, it was shown that different types of data acquisition technologies (smartphone apps or web-based platforms) may have an important effect on patients’ willingness to participate in telehealth programs [31]. Over the years, while there seems to be a greater acceptance of technology among patients compared to the past, the issue of digital patient education still remains a central one. Apps are easy-to-use tools with good penetration and acceptance among the population, and our survey confirmed this among heart failure patients with CIED as well. Additionally, caregivers appear to play a fundamental role. Of note, caregivers greatly increased the penetration of app technology in our population, often being frequent and expert users of apps who were also willing to invest time in patient care (in the same way as the patient would). This finding is concordant with the literature reporting the fundamental role of caregivers in the pathway and outcomes of heart failure patients [32,33].

### 4.4. Patient Adherence to the Use of the App

The second part of the project sought to evaluate the adherence of patients who downloaded the app to collect and report their heart failure status. This represents a fundamental issue in this strategy given that, in other settings, apps are known to be used only in the first few days after being downloaded [31,34]. Our study confirmed that adherence to app use is acceptable at the beginning, being about 63%, but that it decreases over time, even though such a decline appears to plateau after the 26th week from download. The reasons for this suboptimal level of adherence and its decrease over time can be sought in the 30% of subjects who accepted the app despite not being confident with smartphone and app technology, together with the fact that, in most cases, no feedbacks were provided when clinical actions were not deemed necessary on the basis of reviewed CIED and app information (patients declaring to be asymptomatic or adherent to the prescribed therapy). In accordance with previous studies [35], we found that caregiver assistance can improve patient adherence to app use. Furthermore, our project confirmed that in hospitals where dedicated staff are available to educate and encourage patients/caregiver to use the app, adherence is higher [32]. These results therefore suggest that the patient still has little perception of the concept of digital therapy and that a long training process is necessary.

One of the main results of this research project, which, to our knowledge, has not been reported in literature, is that when analyzing the profile of patients who did not agree to receive the app, they were older than 60 years, as reported in Table 1, and had a lower school qualification (77%). As these features also characterized the subgroups of patients showing a higher adherence to the use of the app (Figure 3), we can speculate that patients who appear to be less inclined to receive the app can have better adherence once they accept it than those who seem more open to accept it.

A second point of interest is that more symptomatic (NYHA III–IV) patients have greater adherence (66.0% vs. 46.0; *p* < 0.001), although all the patients received the same instructions from the hospital staff to send at least one diary every week. This may be due to the fact that symptomatic patients, as well as those with lower quality of life, as shown by Sohn et. al. [15], see more benefit in the use of the app compared to the asymptomatic ones, who may instead forget about it if they do not receive any reminders from the app. During this first phase of the Angels of HF project, automatic reminders were not available. A new version of the app, which is also compatible with Huawei devices and allows automatic weekly reminders, has been released after the finalization of the present analysis. The impact of automatic reminders on patient acceptance and adherence to using the app is among the objectives of the second phase of the project that is currently ongoing.

### 4.5. Angels of HF Project’s Next Steps

The next Angels of HF project phase will be aimed at assessing the potential correlation between the CIED HF score and HF patient-reported symptoms, signs, or poor therapy adherence, and, in the long-term, the impact of the use of the CIED HF score integrated with HF-dedicated app information on clinical decisions and ultimately on patient outcomes. The deployment of digital tools for the management of heart failure still requires extensive research to assess their efficacy and patient training to increase awareness and empowerment. Nonetheless, as highlighted in this project, this novel strategy appears to hold a far-reaching potential to reduce scheduled in-office visits or unnecessary medical contacts, thereby maintaining a high level of heart failure management and optimizing healthcare resources [36]. However, similarly to what occurs for remote monitoring [37] and many currently available digital tools [38,39], an extensive implementation in daily practice of apps coupled with CIED diagnostics will require appropriate reimbursement practices targeted at adoption of new models for care delivery in HF patients.

## 5. Limitations

We performed an analysis to evaluate the penetration of app technology in a specific group of patients implanted with a CIED and suffering from HF to understand if this technology may have a role in the remote management of this specific patient population by increasing the amount of information available to physicians for decision making in clinical practice. We do not know if these results may also be extended to HF patients not implanted with a CIED. Due to the availability of an HF-dedicated patient app limited to Medtronic CIEDs, our multicenter project focused on HF patients wearing a CIED from this manufacturer. We cannot exclude that characteristics of patients treated with CIEDs from other companies could slightly differ and influence the survey answers and adherence. Although the survey was administered to a sample of consecutive HF patients presenting at the cardiology departments for follow-up during a specific period, we cannot completely rule out selection bias. However, as patient characteristics were like those of other real-world registries on the HF population with CIED [40,41], we believe that selection bias was limited. Moreover, even if the survey was submitted only to patients referring to 10 Italian hospitals, the differences in terms of the hospitals’ capacity, organization, and geographical distribution makes us more confident about the fact that this population may be a good representative sample of the Italian HF population implanted with a CIED. We cannot exclude some bias due to the presence of a caregiver for some patients. However, this allowed us to evaluate the use of the app in the real world. Any change of caregiver during follow-up was not considered in the assessment of adherence to the app usage. However, we can expect a low frequency of caregiver changes considering that about 60% of caregivers in our observation were cohabiting caregivers.

## 6. Conclusions

This project highlights that a high percentage of heart failure patients are in favor of using an app for better management of their heart failure status. The use of smartphone app technology may be included in the remote management of patients to collect information on worsening of heart failure sign/symptoms and prevent hospitalizations. Younger patients with higher school qualifications are more likely to accept the technology, despite being less diligent in using it, and thus represent the patient group where adherence can be most improved. Older patients with higher NYHA class are more motivated in using the app, thereby feeling connected with their physician. Caregivers are confirmed to have a fundamental role in increasing the penetration of this technology and in improving patient adherence. These data provide a compelling case for the need for specific training of patients and caregivers on the skills required to use app technology for remote patient monitoring as well as to raise awareness on its potential benefits. At the same time, dedicated clinical trials on larger populations over longer follow-up periods are needed to demonstrate how apps can improve the management of patients with heart failure in the long term.

## Figures and Tables

**Table 1:** Baseline patient characteristics and medications.

Variable	Summary Statistics	Total *N* = 495	App *N* = 311	No App *N* = 184	*p*-Value
DEMOGRAPHICS					
Age at first implant (years)	Mean ± SD	66.8 ± 13.1	64.9 ± 14.0	70.2 ± 10.5	<0.001
Sex (male)	% (n/Pts)	78.6 (389/495)	79.1 (246/311)	77.7(143/184)	0.802
MEDICAL HISTORY					
NYHA 3/4	% (n/Pts)	26.2 (118/450)	25.0 (71/284)	28.3 (47/166)	0.441
VT/VF	% (n/Pts)	26.0 (109/419)	27.2 (76/279)	23.6 (33/140)	0.419
AT/AF	% (n/Pts)	33.9 (158/466)	31.6 (93/294)	37.8 (65/172)	0.175
Ischemic cardiopathy	% (n/Pts)	48.5 (230/474)	47.3 (142/300)	50.6 (88/174)	0.496
First-grade AV block	% (n/Pts)	4.0 (20/495)	2.9% (9/311)	6.0 (11/184)	0.092
Second-grade AV block	% (n/Pts)	2.2 (11/495)	1.6 (5/311)	3.3 (6/184)	0.228
Third-grade AV block	% (n/Pts)	5.5 (27/495)	5.8 (18/311)	4.9 (9/184)	0.671
RBBB	% (n/Pts)	3.2 (16/495)	1.0 (3/311)	7.1 (13/184)	<0.001
LBBB	% (n/Pts)	41.4 (205/495)	41.5% (129/311)	41.3 (76/184)	0.970
Left hemiblock	% (n/Pts)	1.8 (9/495)	1.3 (4/311)	2.7 (5/184)	0.249
SND	% (n/Pts)	8.0 (26/327)	8.7 (19/218)	6.4 (7/109)	0.470
History of stroke/TIA	% (n/Pts)	4.1 (17/413)	4.0 (11/275)	4.3 (6/138)	0.867
Hypertension	% (n/Pts)	64.2 (278/433)	64.5 (187/290)	63.6 (91/143)	0.863
Diabetes	% (n/Pts)	30.1 (126/419)	30.0 (85/283)	30.1 (41/136)	0.981
Chronic kidney disease	% (n/Pts)	21.0 (85/404)	18.8 (51/271)	25.6 (34/133)	0.118
COPD	% (n/Pts)	10.4 (42/404)	11.1 (30/270)	9.0 (12/134)	0.504
CHA₂DS₂-VASc ≥ 4	% (n/Pts)	41.9 (126/301)	41.1 (85/207)	43.6 (41/94)	0.677
BASELINE ASSESSMENT					
Intrinsic QRS (ms)	Mean ± SD	138.3 ± 29.5	134.8 ± 29.4	144.6 ± 28.7	0.006
LVEF (%)	Mean ± SD	34.9 ± 10.9	34.6 ± 10.4	35.3 ± 11.7	0.937
DEVICE TYPE					
CRT-D	% (n/Pts)	52.7 (259/491)	54.7 (169/309)	49.5 (90/182)	0.371
CRT-P	% (n/Pts)	7.7 (38/491)	7.1 (22/309)	8.8 (16/182)	
DC-ICD	% (n/Pts)	17.3 (85/491)	17.8 (55/309)	16.5 (30/182)	
SC-ICD	% (n/Pts)	21.4 (105/491)	20.1 (62/309)	23.6 (43/182)	
IPG	% (n/Pts)	0.8 (4/491)	0.3 (1/309)	1.6 (3/182)	
BASELINE MEDICATIONS					
Beta-blocker	% (n/Pts)	75.1 (293/390)	77.4 (199/257)	70.7 (94/133)	0.143
ACE inhibitor/ARBs	% (n/Pts)	58.2 (228/392)	56.4 (146/259)	61.7 (82/133)	0.315
Diuretic	% (n/Pts)	76.1 (309/406)	76.8 (208/271)	74.8 (101/135)	0.666
ARNi	% (n/Pts)	23.0 (70/305)	24.8 (53/214)	18.7 (17/91)	0.248
Antiplatelet	% (n/Pts)	47.0 (186/396)	46.7 (122/261)	47.4 (64/135)	0.900
OAC	% (n/Pts)	45.8 (164/358)	42.6 (103/242)	52.6 (61/116)	0.075

NYHA = New York Heart Association; VT = ventricular tachycardia; VF = ventricular fibrillation; AT = atrial tachycardia; AF = atrial fibrillation; AV = atrioventricular; LBBB = left bundle branch block; RBBB = right bundle branch block; SND = sinus node dysfunction; TIA = transient ischemic attack; COPD = chronic obstructive pulmonary disease; CRT-D = cardiac resynchronization therapy defibrillator; CRT-P = cardiac resynchronization therapy pacemaker; DC-ICD = dual-chamber implanter cardioverter defibrillator; SC-ICD = single-chamber implantable cardioverter defibrillator; IPG = implantable pulse generator; LVEF = left ventricular ejection fraction; ACE = angiotensin-converting enzyme; ARB = angiotensin receptor blocker; ARNi = angiotensin receptor–neprilysin inhibitor; OAC = oral anticoagulation.

**Table 2:** Comparison of key findings of the Angels of HF project with the results of prior work in the field of smartphone/tablet app usage for HF management.

	N Subjects	Population	N Sites	Geographies	Type of Investigated Health App	Endpoints	Smartphone/Tablet Access	Able to Use Apps	Using Health Apps	Intention to Use Health App for HF	Adherence in Using app for HF	App Intervention Effect on Patient Outcomes	Other Results
**ANGELS OF HF PROJECT**													
Ziacchi M et al.	495	HF patients with cardiac implantable electronic devices: - Mean age: 67 y; - 21% women; - 42% high school or higher education.	10	Italy	App with remote HF data monitored by nurse/cardiologist + clinical feedback/intervention, if needed	Patient ownership/access to smartphone/tablet; Patients’ or caregivers’ acceptance to receive an HF-dedicated app; Patient adherence to using the HF-dedicated app during the first year of usage.	60% on patient smartphone/ tablet; 24% on caregiver smartphone/tablet.	69%	21%	85%	49% (mean adherence at 1 year).	Not evaluated yet.	Younger patients with higher school qualifications are more likely to accept the technology, despite being less diligent in using it. The caregiver has a fundamental role in increasing the penetration of apps and in improving patient adherence.
**PREVIOUS STUDIES**													
Leigh JW et al. [13]	100	HF patients: - Mean age: 61 y - 63% women; - 79% non-White ethnicity - 82% high school or higher education.	1	US	HF self-care app	Smartphone ownership and patient attitudes toward using a health app for HF.	68%	60%	22%	N.A.	N.A.	N.A.	Ethnic minorities had higher smartphone ownership rates compared with White patients with HF. Moderate significant association between smartphone ownership and age, education, and employment status.
Cajita MI et al. [14]	129	HF patients ≥65 y: - Mean age: 71 y - 26% women - 43% non-White ethnicity. - >79% high school or higher education	1	US	HF self-care app	Factors that influence intention to use a health app for HF; current smartphone use; intention to use a health app if recommended by cardiologist.	57.4%	N.A.	N.A.	85%	N.A.	N.A.	Social influence and higher perceived ease of use and usefulness were both associated with higher intention to use a health app. Perceived financial cost and eHealth literacy were not significantly associated with intention to use mHealth.
Sohn A et al. [15]	49	HF patients between 50-80 y: - Mean age: 64 y - 32% women - 33% non-White ethnicity - 100% high school or higher education.	1	US	HF self-care app	Interest in a smartphone app for HF; Determine factors that influence patient interest in app for HF.	90%	N.A.	N.A.	79%	N.A.	N.A.	Age correlated negatively with interest in activity tracking, HF symptoms management tips, and reminder features of the app for HF.
Shan R et al. [16]	1903	Patients with CV disease or CV risk factors: - 30% ≥65 y; 59% 36–65 y; - 46% women; - 37% non-White ethnicity; - 88% high school or higher education.	National Survey	US	All types	Prevalence of health app access and usage; Association between CV disease risk and health app uptake.	73%	N.A.	48%	N.A.	N.A.	N.A.	CVD risk was associated with sharing information from smartphone with a clinician.
Boriani et al. [17]	1067	Cardiology outpatients: - Mean age: 70 y - 41% women - 68% secondary school or higher education.	1	Italy	App for teleconference	Evaluate digital literacy among cardiology outpatients to assess the possibilities to extend telemedicine/televisits during COVID pandemic.	59%	57%	N.A.	N.A.	N.A.	N.A.	The most used devices for internet access were smartphones, and WhatsApp represented the most used app. Internet users were younger compared to those who did not use the internet and had a higher educational level.

## Data Availability

The data presented in this manuscript are available on request from the corresponding author. The data are not publicly available due to privacy policy restrictions of hospitals involved in the project.

## Appendix A

English translation of the patient survey.

1. Does the patient have a smartphone/tablet?
- Yes, the patient has a smartphone/tablet
- No, but the patient lives with someone who has a smartphone/tablet (cohabiting caregiver)
- No, but the patient often hangs out with someone who has a smartphone/tablet (non-cohabiting caregiver)
- No, and the patient does not hang out with anyone who has a smartphone/tablet
2. If no, why not?
- The patient does not know what a smartphone/tablet is
- The patient considers a smartphone/tablet unnecessary
- The patient is not used to this kind of technology
- The patient does not want to spend money for this kind of device
- The patient does not want to spend money for browsing the internet
- Other
3. Which is the patient’s (or caregiver’s, if the patient does not have a smartphone) school qualification?
- Primary school
- Lower secondary school
- High school
- Bachelor’s/master’s degree
- Other
4. Does the patient/caregiver usually browse the internet through the smartphone/tablet?
- Yes, often
- Yes, sometimes
- No
5. How long has the patient/caregiver been having a smartphone /tablet?
- Less than 6 months
- Between 6 and 12 months
- Between 1 and 2 years
- Between 2 and 5 years
- More than 5 years
6. The patient/caregiver is able to perform the following actions through the smartphone/tablet:
- Make a phone call
- Send a text message/take a picture
- Send emails
- Install apps
- Use apps
- Browse the internet
7. Has the patient/caregiver ever used health apps on their smartphone/tablet?
- Yes
- No
8. Would the patient/caregiver be willing to send this kind of information through a FREE app for their smartphone/tablet on a weekly basis?
- Yes
- Yes, but the patient/caregiver is not sure he/she can find anyone able to help them in doing it
- Yes, but the patient/caregiver is afraid to forget to do it
- No, the patient/caregiver is afraid about possible data privacy violation
- No, the patient/caregiver does not have time to do it
- No, for other reason
9. How much time would the patient/caregiver be willing to dedicate to this activity?
- 5 min, every day
- 5 min for 2 times a week
- 5 min for 1 time a week
10. How much time would the patient/caregiver be willing to dedicate to this activity?
- 5 min, every day
- 5 min for 2 times a week
- 5 min for 1 time a week

## References

[1] McDonagh T.A., Metra M., Adamo M., Gardner R.S., Baumbach A., Böhm M., Burri H., Butler J., Čelutkienė J., Chioncel O. (2022). 2021 ESC Guidelines for the diagnosis and treatment of acute and chronic heart failure: Developed by the Task Force for the diagnosis and treatment of acute and chronic heart failure of the European Society of Cardiology (ESC) with the special contribution of the Heart Failure Association (HFA) of the ESC. Eur. J. Heart Fail..

[2] Russo M.J., Gelijns A.C., Stevenson L.W., Sampat B., Aaronson K.D., Renlund D.G., Ascheim D.D., Hong K.N., Oz M.C., Moskowitz A.J. (2008). The cost of medical management in advanced heart failure during the final two years of life. J. Card. Fail..

[3] Jencks S.F., Williams M.V., Coleman E.A. (2009). Rehospitalizations among patients in the Medicare fee-for-service program. N. Engl. J. Med..

[4] Adamson P.B. (2009). Pathophysiology of the transition from chronic compensated and acute decompensated heart failure: New insights from continuous monitoring devices. Curr. Heart Fail. Rep..

[5] Whellan D.J., Ousdigian K.T., Al-Khatib S.M., Pu W., Sarkar S., Porter C.B., Pavri B.B., O’Connor C.M. (2010). Combined heart failure device diagnostics identify patients at higher risk of subsequent heart failure hospitalizations: Results from PARTNERS HF (Program to Access and Review Trending Information and Evaluate Correlation to Symptoms in Patients with Heart Failure) study. J. Am. Coll. Cardiol..

[6] Boriani G., Da Costa A., Ricci R.P., Quesada A., Favale S., Iacopino S., Romeo F., Risi A., Stefano L.M.d.S., Navarro X. (2013). The monitoring resynchronization devices and cardiac patients (MORE-CARE) randomized controlled trial: Phase 1 results on dynamics of early intervention with remote monitoring. J. Med. Internet Res..

[7] Cowie M.R., Sarkar S., Koehler J., Whellan D.J., Crossley G.H., Tang W.H.W., Abraham W.T., Sharma V., Santini M. (2013). Development and validation of an integrated diagnostic algorithm derived from parameters monitored in implantable devices for identifying patients at risk for heart failure hospitalization in an ambulatory setting. Eur. Heart J..

[8] Gula L.J., Wells G.A., Yee R., Koehler J., Sarkar S., Sharma V., Skanes A.C., Sapp J.L., Redfearn D.P., Manlucu J. (2014). A novel algorithm to assess risk of heart failure exacerbation using ICD diagnostics: Validation from RAFT. Heart Rhythm..

[9] Burri H., da Costa A., Quesada A., Ricci R.P., Favale S., Clementy N., Boscolo G., Villalobos F.S., Stefano L.M.d.S., Sharma V. (2018). Risk stratification of cardiovascular and heart failure hospitalizations using integrated device diagnostics in patients with a cardiac resynchronization therapy defibrillator. Europace.

[10] Sammut-Powell C., Taylor J.K., Motwani M., Leonard C.M., Martin G.P., Ahmed F.Z. (2022). Remotely Monitored Cardiac Implantable Electronic Device Data Predict All-Cause and Cardiovascular Unplanned Hospitalization. J. Am. Heart Assoc..

[11] Ahmed F.Z., Sammut-Powell C., Kwok C.S., Tay T., Motwani M., Martin G.P., Taylor J.K. (2022). Remote monitoring data from cardiac implantable electronic devices predicts all-cause mortality. Europace.

[12] Seto E., Leonard K.J., Cafazzo J.A., Barnsley J., Masino C., Ross H.J. (2012). Mobile phone-based telemonitoring for heart failure management: A randomized controlled trial. J. Med. Internet Res..

[13] Leigh J.W., Gerber B.S., Gans C.P., Kansal M.M., Kitsiou S. (2022). Smartphone Ownership and Interest in Mobile Health Technologies for Self-care among Patients with Chronic Heart Failure: Cross-sectional Survey Study. JMIR Cardio.

[14] Cajita M.I., Hodgson N.A., Budhathoki C., Han H.R. (2017). Intention to Use mHealth in Older Adults with Heart Failure. J. Cardiovasc. Nurs..

[15] Sohn A., Speier W., Lan E., Aoki K., Fonarow G., Ong M., Arnold C. (2019). Assessment of Heart Failure Patients’ Interest in Mobile Health Apps for Self-Care: Survey Study. JMIR Cardio.

[16] Shan R., Ding J., Plante T.B., Martin S.S. (2019). Mobile Health Access and Use Among Individuals with or At Risk for Cardiovascular Disease: 2018 Health Information National Trends Survey (HINTS). J. Am. Heart Assoc..

[17] Boriani G., Maisano A., Bonini N., Albini A., Imberti J.F., Venturelli A., Menozzi M., Ziveri V., Morgante V., Camaioni G. (2019). Digital literacy as a potential barrier to implementation of cardiology tele-visits after COVID-19 pandemic: The INFO-COVID survey. J. Am. Heart Assoc..

[18] Anugu P., Ansari A.Y., Min Y.-I., Benjamin E.J., Murabito J., Winters K., Turner E., Correa A. (2022). Digital Connectedness in the Jackson Heart Study: Cross-sectional Study. J. Med. Internet Res..

[19] Kitsiou S., Vatani H., Paré G., Gerber B.S., Buchholz S.W., Kansal M.M., Leigh J., Creber R.M.M. (2021). Effectiveness of Mobile Health Technology Interventions for Patients with Heart Failure: Systematic Review and Meta-analysis. Can. J. Cardiol..

[20] Vuorinen A.L., LeppÃnen J., Kaijanranta H., Kulju M., Heliö T., van Gils M., LÃhteenmÃki J. (2014). Use of Home Telemonitoring to Support Multidisciplinary Care of Heart Failure Patients in Finland: Randomized Controlled Trial. J. Med. Internet Res..

[21] Yanicelli L.M., Goy C.B., González V.d.C., Palacios G.N., Martínez E.C., Herrera M.C. (2021). Non-invasive home telemonitoring system for heart failure patients: A randomized clinical trial. J. Telemed. Telecare.

[22] Mobile Fact Sheet (2021). Pew Research Center. https://www.pewresearch.org/internet/fact-sheet/mobile/.

[23] The Mobile Economy Europe 2022 Report by GSMA. https://www.gsma.com/mobileeconomy/europe/.

[24] Gual-Montolio P., Suso-Ribera C., García-Palacios A., Castilla D., Zaragoza I., Bretón-López J. (2023). Enhancing Internet-based psychotherapy for adults with emotional disorders using ecological momentary assessments and interventions: Study protocol of a feasibility trial with “My EMI, Emotional Well-being” app. Internet Interv..

[25] Shaw J., Acharya C., Albhaisi S., Fagan A., McGeorge S., White M.B., Lachar J., Olson J., Olofson A., Bergstrom L. (2023). Subjective and objective burden on providers from a multicenter app-based study of patients with cirrhosis and caregivers. Hepatol. Commun..

[26] Bhuyan S.S., Lu N., Chandak A., Kim H., Wyant D., Bhatt J., Kedia S., Chang C.F. (2016). Use of mobile health applications for health-seeking behavior among US adults. J. Med. Syst..

[27] Shuren J., Patel B., Gottlieb S. (2018). FDA Regulation of Mobile Medical Apps. JAMA.

[28] Melenhorst A.-S., Rogers W.A., Bouwhuis D.G. (2006). Older adults’ motivated choice for technological innovation: Evidence for benefit-driven selectivity. Psychol. Aging.

[29] Mitzner T.L., Boron J.B., Fausset C.B., Adams A.E., Charness N., Czaja S.J., Dijkstra K., Fisk A.D., Rogers W.A., Sharit J. (2010). Older Adults Talk Technology: Technology Usage and Attitudes. Comput. Hum. Behav..

[30] Liu S., Li J., Wan D.Y., Li R., Qu Z., Hu Y., Liu J. (2022). Effectiveness of eHealth Self-management Interventions in Patients with Heart Failure: Systematic Review and Meta-analysis. J. Med. Internet Res..

[31] Schreier G., Eckmann H., Hayn D., Kreiner K., Kastner P., Lovell N. (2012). Web versus App—Compliance of patients in a telehealth diabetes management programme using two different technologies. J. Telemed. Telecare.

[32] Thodi M., Bistola V., Lambrinou E., Keramida K., Nikolopoulos P., Parissis J., Farmakis D., Filippatos G. (2022). A randomized trial of a nurse-led educational intervention in patients with heart failure and their caregivers: Impact on caregiver outcomes. Eur. J. Cardiovasc. Nurs..

[33] Purcell C., Purvis A., Cleland J.G.F., Cowie A., Dalal H.M., Ibbotson T., Murphy C., Taylor R.S. (2023). Home-based cardiac rehabilitation for people with heart failure and their caregivers: A mixed-methods analysis of the roll out an evidence-based programme in Scotland (SCOT:REACH-HF study). Eur. J. Cardiovasc. Nurs..

[34] Shih P.C., Han K., Poole E.S., Rosson M.B., Carroll J.M. (2015). Use and Adoption Challenges of Wearable Activity Trackers. iConference 2015 Proceedings.

[35] Clements L., Frazier S.K., Lennie T.A., Chung M.L., Moser D.K. (2022). Improvement in Heart Failure Self-Care and Patient Readmissions with Caregiver Education: A Randomized Controlled Trial. West. J. Nurs. Res..

[36] Sgreccia D., Mauro E., Vitolo M., Manicardi M., Valenti A.C., Imberti J.F., Ziacchi M., Borini G. (2022). Implantable cardioverter defibrillators and devices for cardiac resynchronization therapy: What perspective for patients’ apps combined with remote monitoring?. Expert. Rev. Med. Devices.

[37] Boriani G., Burri H., Svennberg E., Imberti J.F., Merino J.L., Leclercq C. (2022). Current status of reimbursement practices for remote monitoring of cardiac implantable electrical devices across Europe. Europace.

[38] Boriani G., Svennberg E., Guerra F., Linz D., Casado-Arroyo R., Malaczynska-Rajpold K., Duncker D., Boveda S., Merino J.L., Leclercq C. (2022). Reimbursement practices for use of digital devices in atrial fibrillation and other arrhythmias: A European Heart Rhythm Association survey. Europace.

[39] Svennberg E., Tjong F., Goette A., Akoum N., Di Biase L., Bordachar P., Boriani G., Burri H., Conte G., Deharo J.C. (2022). How to use digital devices to detect and manage arrhythmias: An EHRA practical guide. Europace.

[40] Bonhorst D., Guerreiro S., Fonseca C., Cardim N., Macedo F., Adragão P. (2019). Real-life data on heart failure before and after implantation of resynchronization and/or defibrillation devices—The Síncrone study. Rev. Port. Cardiol..

[41] Raafs A.G., Linssen G.C., Brugts J.J., Erol-Yilmaz A., Plomp J., Smits J.P., Nagelsmit M.J., Oortman R.M., Hoes A.W., Brunner-LaRocca H. (2020). Contemporary use of devices in chronic heart failure in the Netherlands. ESC Heart Fail..

